# Incidental finding of tuberous sclerosis complex in a woman with hematuria: A case report of renal angiomyolipoma and review of the literature

**DOI:** 10.1002/ccr3.6913

**Published:** 2023-02-08

**Authors:** Gauri Adhikari, Prabin Pandey, Kishor Bhattarai, Chhabi Khadka, Gopal Adhikari

**Affiliations:** ^1^ Department of Internal Medicine Nepalese Army Institute of Health Sciences‐College of Medicine Kathmandu Nepal; ^2^ Department of Radio‐diagnosis and Imaging National Academy of Medical Sciences Kathmandu Nepal

**Keywords:** angiofibroma, angiomyolipoma, hematuria, radial migration lines, subependymal nodules, tuberous sclerosis complex

## Abstract

Tuberous sclerosis complex (TSC) is a rare genetic multisystem disorder that was first described by Von Recklinghausen. We describe a case of a female, who initially presented with hematuria and was later found to have multiple manifestations of the disease. The report emphasizes the value of investigations on suspected cases.

## Graphical abstract

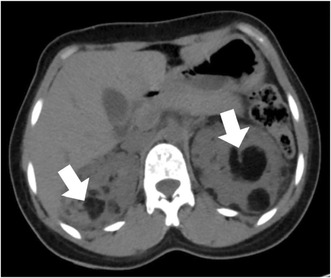

## 1 INTRODUCTION

Tuberous sclerosis (TS) is a rare autosomal dominant multisystem disorder that was described first by Von Recklinghausen and later by Desiree‐Magloire Bourneville.^1^ The condition has a prevalence ranging from 1:6000 to 1:10000 live births and a population prevalence of 1:20,000.^2^, ^3^ Using the most recent diagnostic criteria, a study in Germany estimated the disease's incidence rate to range from 1:6760 to 1:13,520 live births.^4^ People of any age group, sex, or ethnic group can be affected by the condition.

Tuberous sclerosis complex 1 (TSC 1) or tuberous sclerosis complex 2 (TSC 2) are two genes linked to the formation of hamartomas in various organs of the body, including the brain, kidneys, skin, lungs, and liver.^1^ Seizures, angiofibroma, and mental retardation are a part of classic triad of TS that is seen in only 29% of patients.^5^ We describe the case of a 26‐year‐old female who presented with a complaint of hematuria in the emergency department of a tertiary care center in Nepal.

### 1.1 Case presentation

A 26‐year‐old married female presented to the emergency department with complaints of bleeding during micturition for 1 week and lower abdominal pain for 5 days. The bleeding was sudden on the onset, painless in nature, and persistent for about a week. It was followed by abdominal pain in the lower region. Her prior medical history, including gynecological history, was insignificant. She was a nonsmoker and nonalcoholic. Her only medication was oral combined contraceptive pills.

**FIGURE 1:**
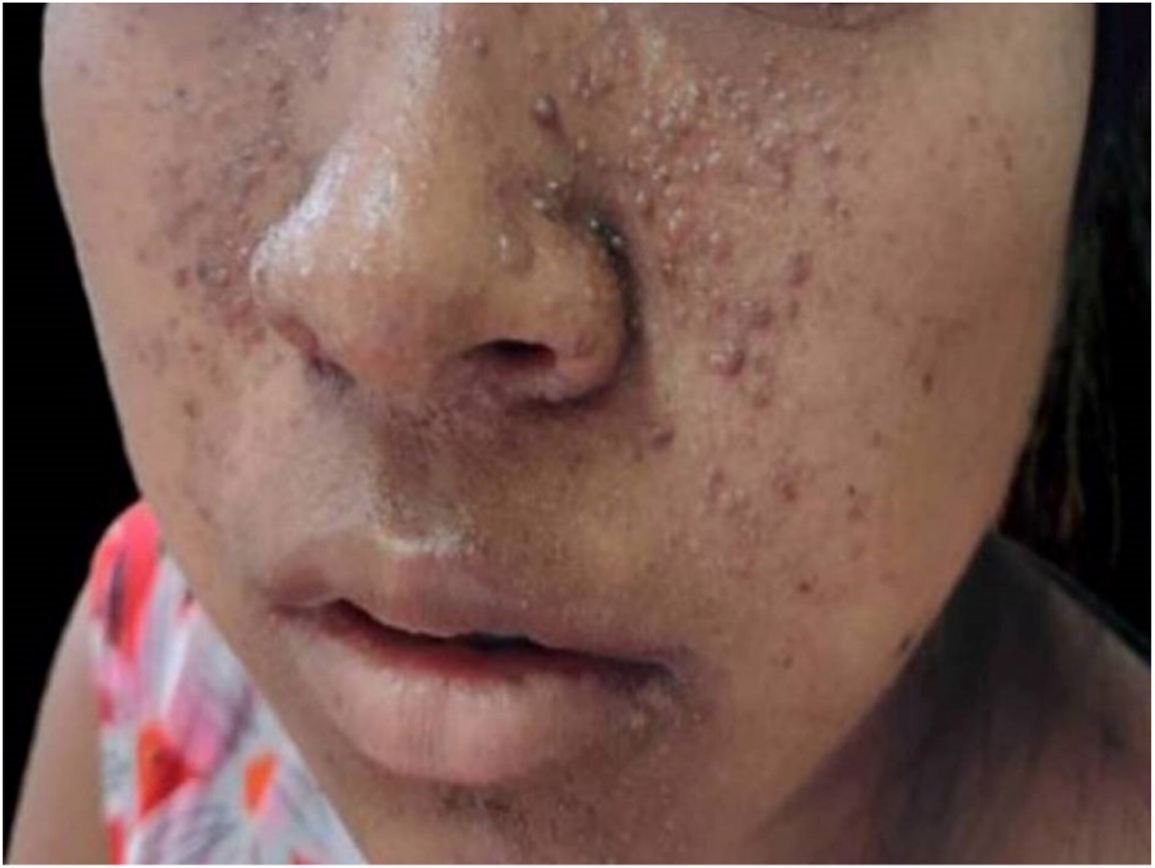
Facial angiofibromas

**FIGURE 2:**
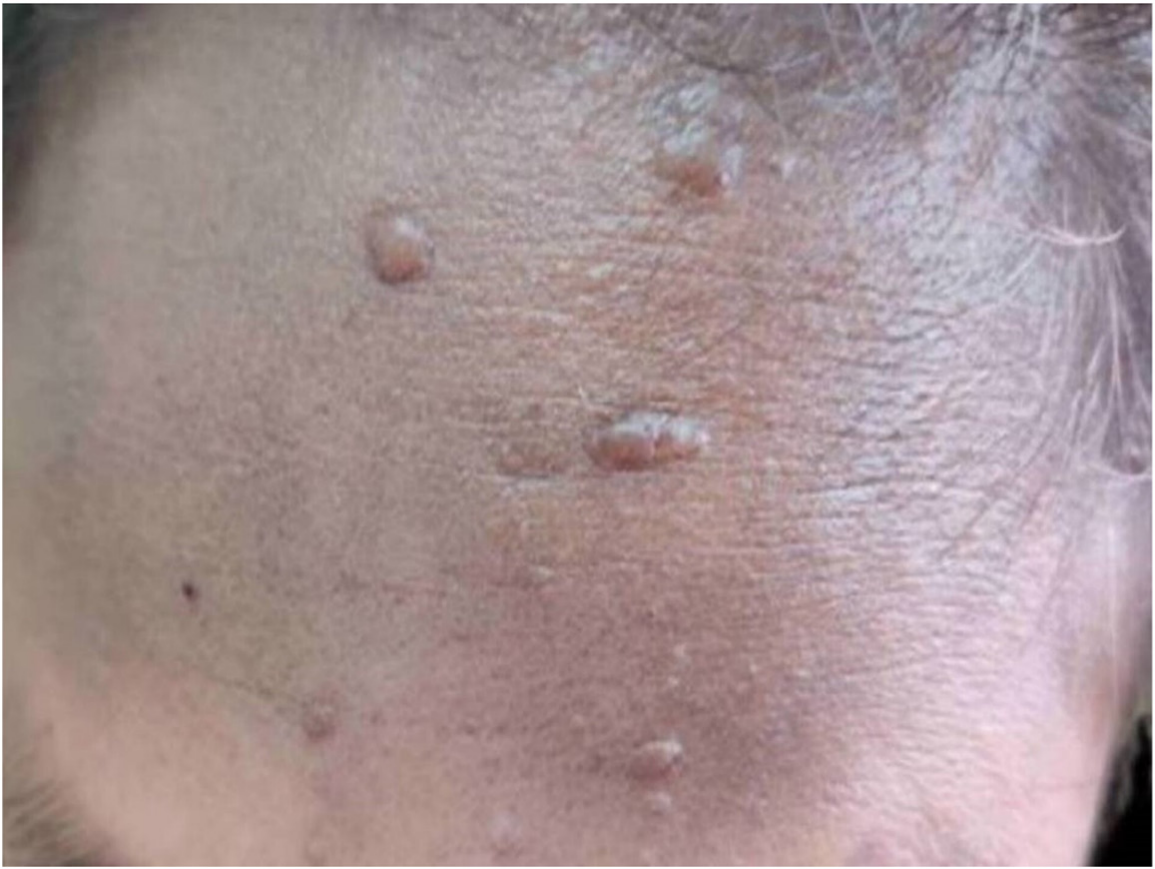
Multiple fibrous plaque on forehead

On examination, she had multiple facial angiofibromas and multiple fibrous plaques on her forehead as shown in Figures 1 and 2. Her organ system examination was found to be normal except for mild suprapubic fullness that was nontender on palpation and dull on percussion.

Her initial hemoglobin was 7.7 gm/dl, and her total count was slightly raised to 12,400/mm^3^. Her other laboratory investigations including electrocardiography, kidney function tests, and liver function tests were essentially normal.

**FIGURE 3:**
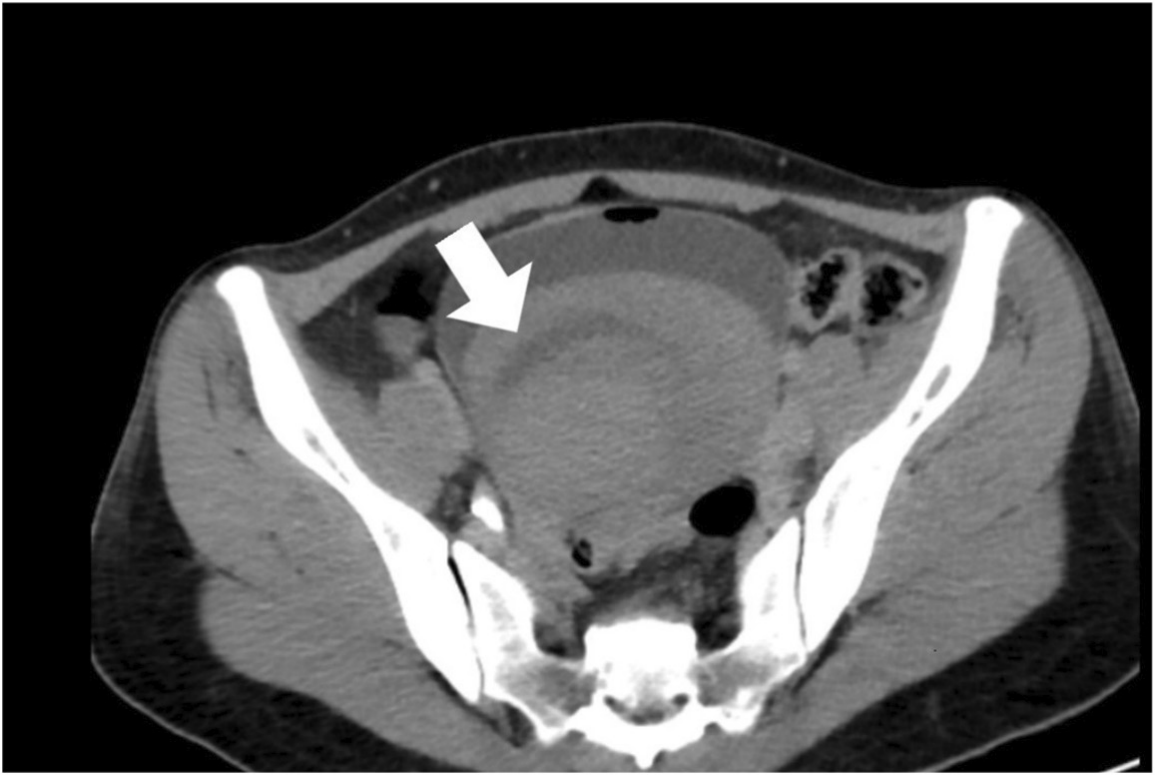
Axial pelvic computed tomography, illustrating urinary bladder with hematoma (white arrow) which was later evacuated with cystoscopy

Her hemoglobin was raised to 9.9 gm/dl after receiving three units of whole blood transfusion. She persistently had hematuria and developed urinary obstruction following which an intravenous urogram with computed tomography (CT) was done which showed a large hyperdense structure in the urinary bladder measuring 12.4 × 12.2 × 11.2 cm and almost filling the lumen as shown in Figure 3.

**FIGURE 4:**
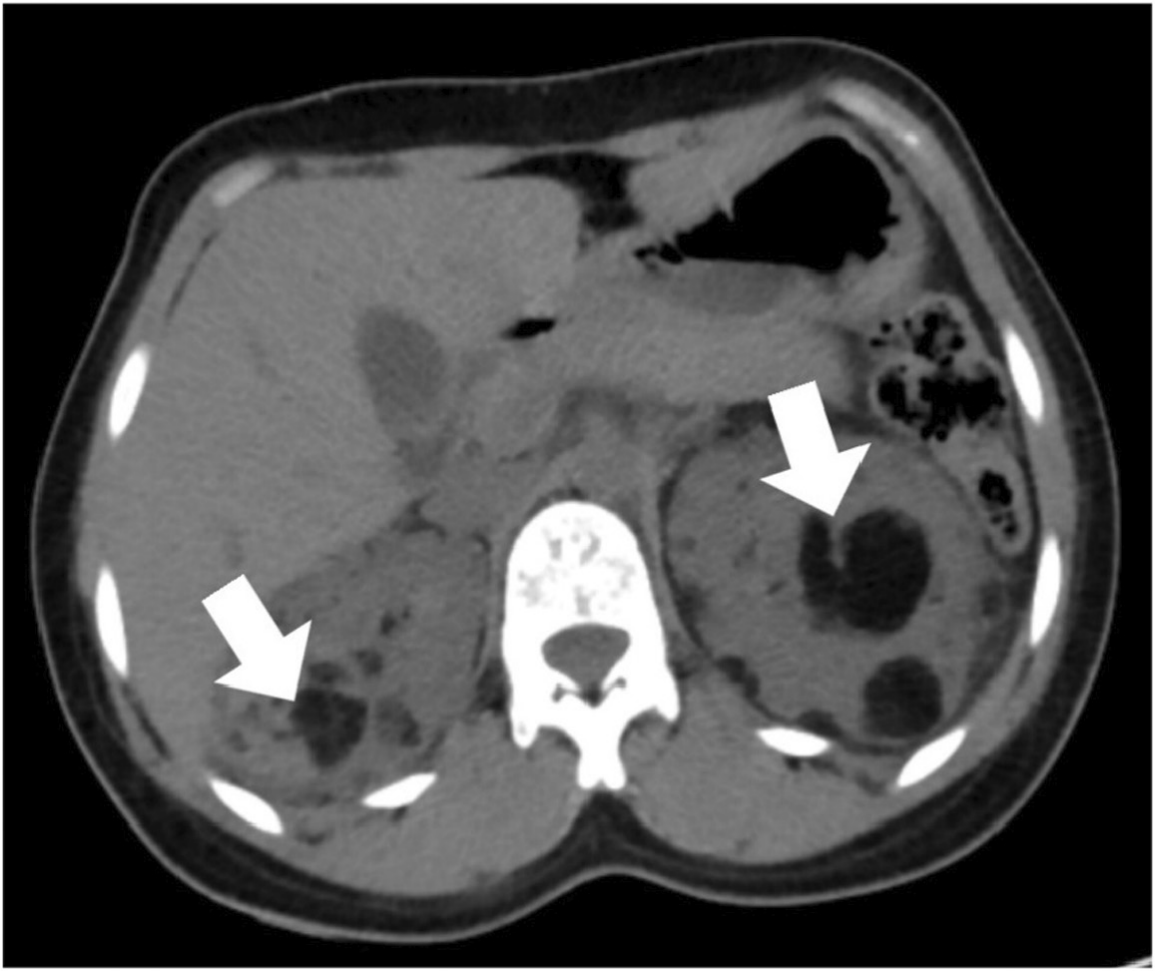
Axial abdominal computed tomography, illustrating multiple bilateral renal angiomyolipoma. White arrow shows darker fat attenuation seen in the lesion

**FIGURE 5:**
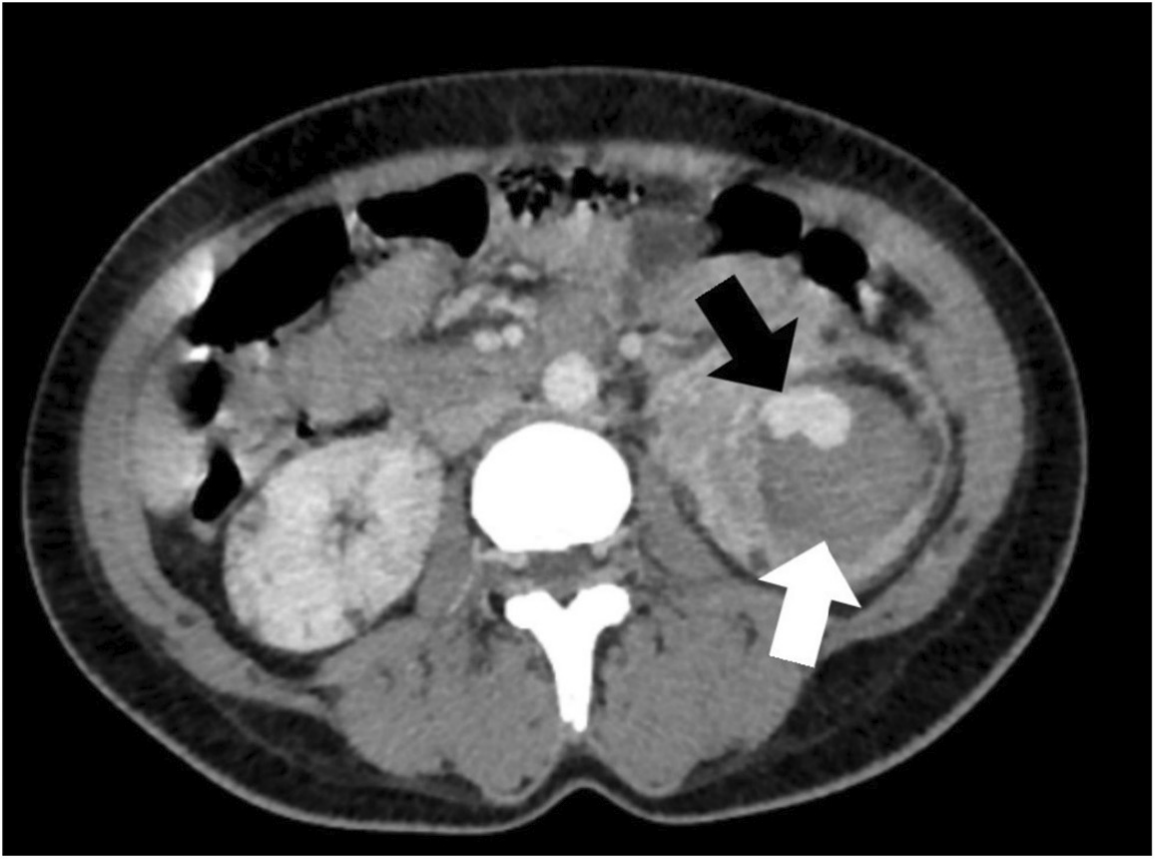
Axial abdominal computed tomography, illustrating aneurysm (black arrow) with adjacent hematoma (white arrow) in the left kidney

**FIGURE 6:**
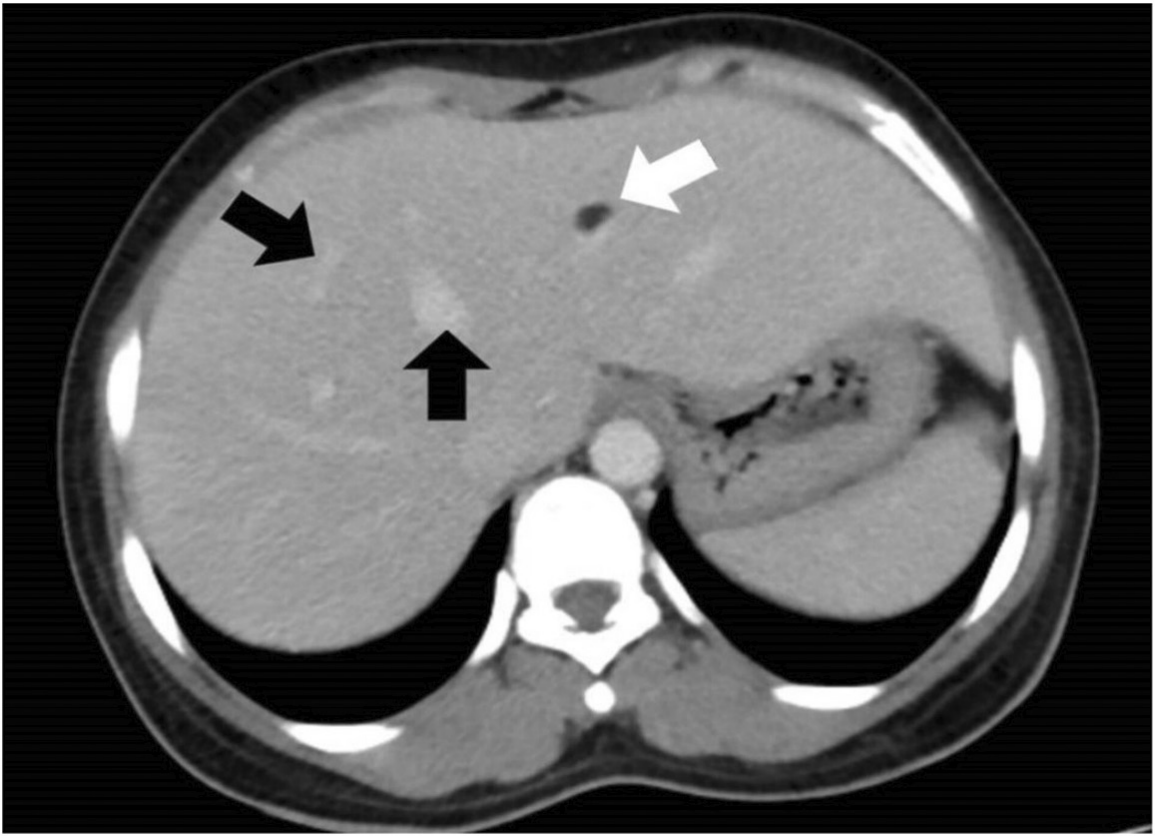
Axial abdominal computed tomography, illustrating fat attenuating lesion (white arrow) with enhancing solid component (black arrow) in the liver

Additionally, multiple fat attenuating lesions with soft tissue components were noted to be randomly distributed in both kidneys as shown in Figure 4. The lesion on the left kidney also demonstrated the presence of an aneurysmal vessel, as shown in Figure 5. Furthermore, multiple well‐defined fat‐attenuating lesions with an enhanced solid component were noted in the liver as shown in Figure 6.

Cystoscopy was done to relieve urinary obstruction, and about 500 ml of organized clot was removed from the urinary bladder. During the procedure, gross blood was visualized at the ureteral meatus.

**FIGURE 7:**
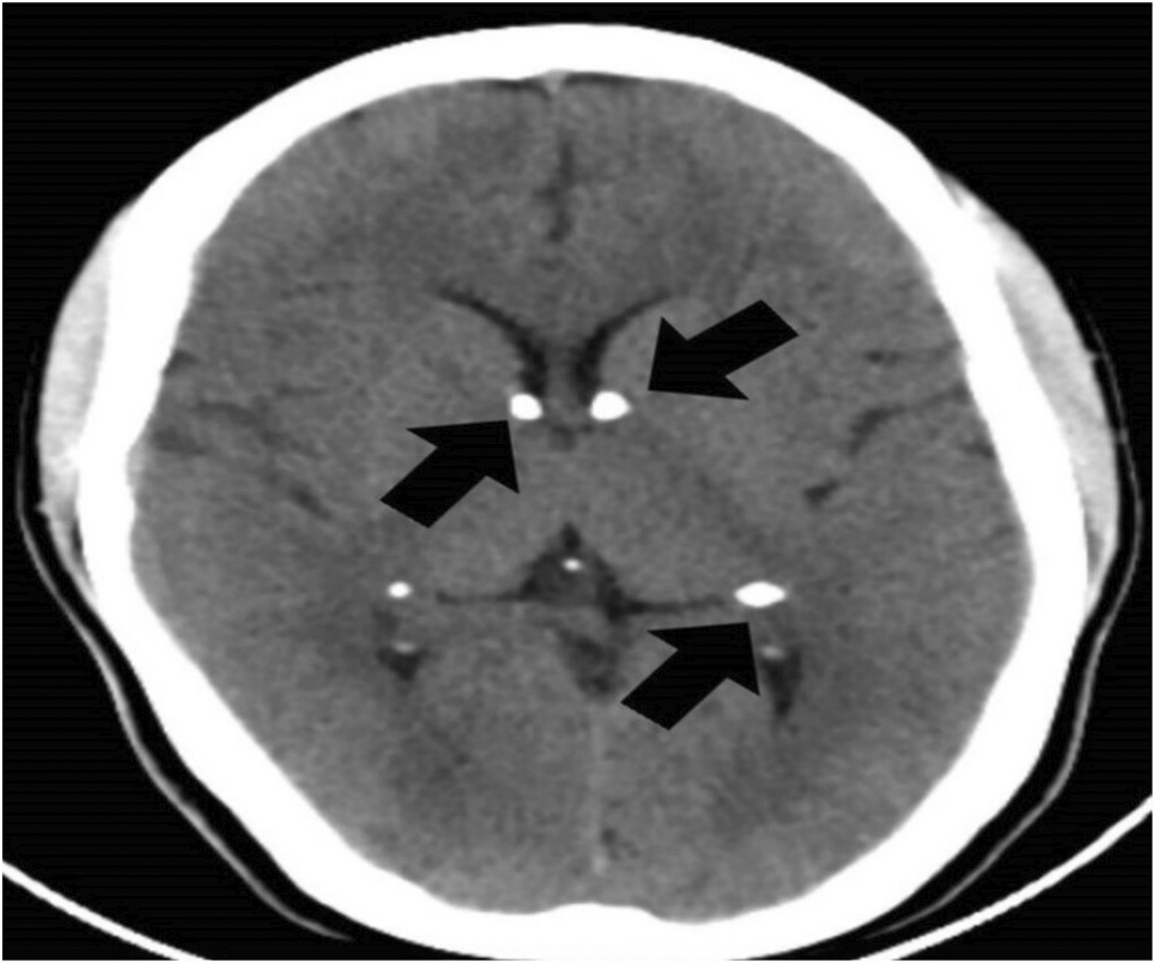
Axial computed tomography of the brain, calcified subependymal nodules along the margin of the ventricles (black arrows)

**FIGURE 8:**
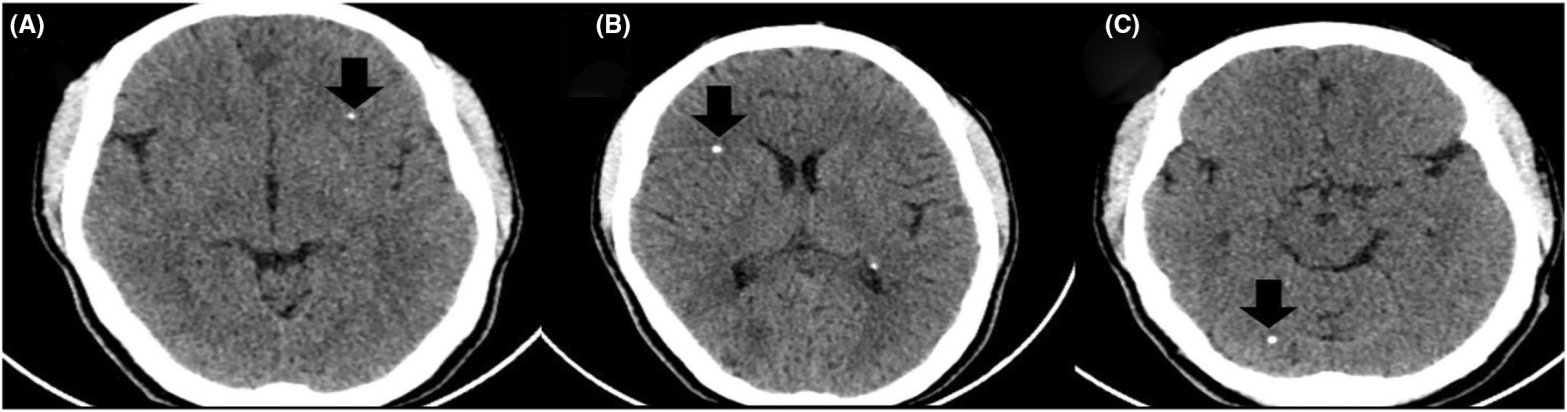
Axial computed tomography of the brain, illustrating calcified cortical dysplasia (black arrows) in the left frontal region (A), right frontal region (B), and right occipital region (C)

**FIGURE 9:**
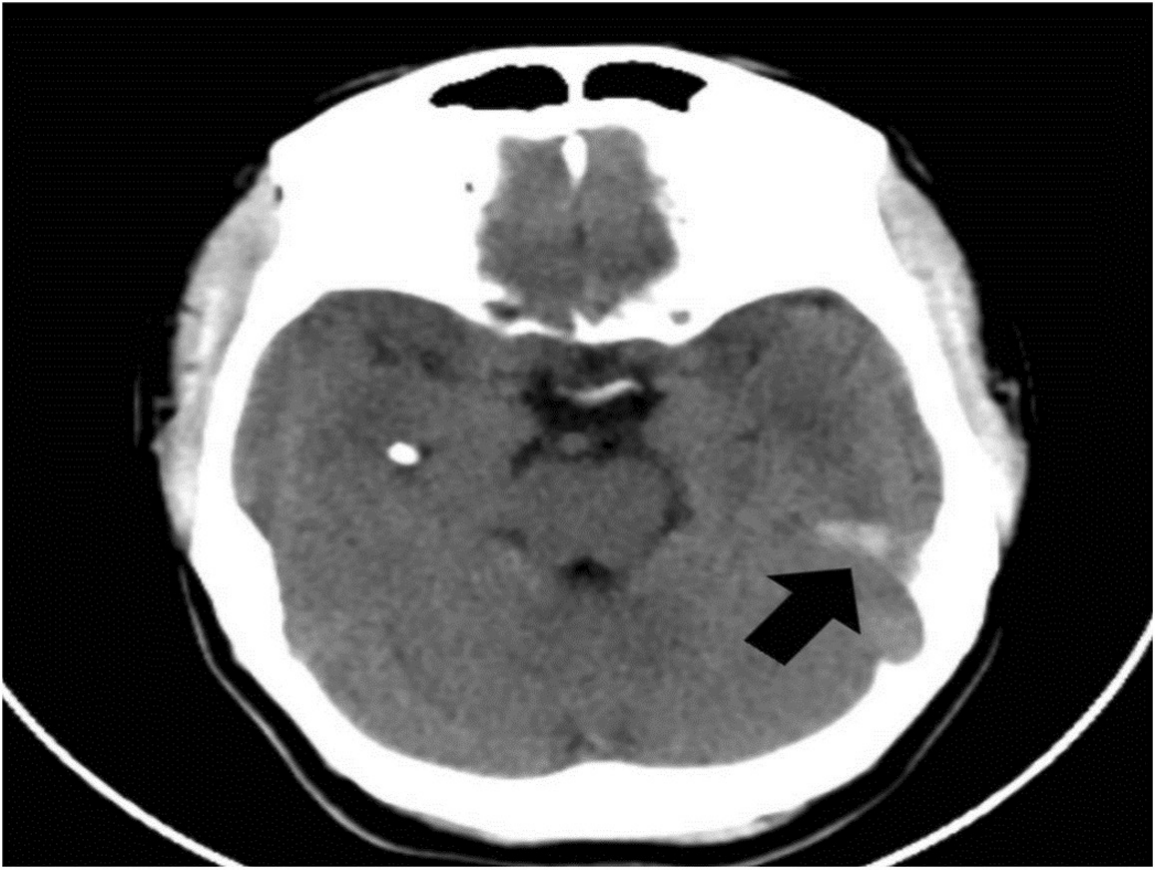
Axial computed tomography of the brain, illustrating cerebral white matter radial migration lines (black arrow)

Considering her findings, a differential diagnosis of TS, smooth muscle hamartomas, neurofibromas, and multiple endocrine neoplasia was made. She received further testing, including a computed tomography scan of her head to help with the diagnosis (the patient refused an MRI). Her brain scan showed multiple calcified nodules along the margin of the ventricles, the largest one measuring 9 × 6 mm (subependymal hamartomas) and also in subcortical regions (subcortical tubers), as shown in Figures 7 and 8, respectively. In addition, her CT image also showed cerebral white matter radial migration lines, which are shown in Figure 9.

Interdepartmental discussion was made, and she was diagnosed as having TSC as she met the diagnostic criteria for definite TSC.^3^ The patient and her family were counseled regarding the condition, the possibility of another bleeding per urethra that might be fatal, and the probable need for nephrectomy. Despite the counseling, she opted for medicinal care with regular screening and follow‐up for her ailment. During discharge, her vital signs were stable, and her hemoglobin was 12.2 mg/dl.

## 2 DISCUSSION

Tuberous sclerosis is a rare inherited neurocutaneous syndrome affecting multiple organ systems that can have many manifestations associated with severe morbidity and potential mortality. TS is due to the mutation of genes encoding hamartin and tuberin, which leads to the uncontrolled growth of hamartomas or tubers in multiple organs of the body. Clinical manifestations of the condition have a varied age predilection, and the extent to which one or more organ systems are involved and severity are also diverse.^3^

Diagnostic criteria given by the International TSC Clinical Consensus Group in 2012 clearly mentioned the importance of independent genetic diagnostic criteria and clinical diagnostic criteria. It is to be noted that in about 10%–15% of the patients meeting the clinical diagnostic criteria, no mutation has been identified by conventional genetic testing.^2^ This shows the importance of other clinical criteria in resource poor settings where genetic testing is difficult to attain for most of the patients. Also, about two‐thirds of the cases are sporadic, which might explain the absence of family history or features suggestive of TS in any member of the family in this patient.^1^

Findings in TS that are part of the clinical diagnostic criteria are represented on Table 1.^1^, ^2^, ^3^

**TABLE 1:** Findings of tuberous sclerosis in different organ system of the body classified as a part of clinical diagnostic criteria

Major criteria	Minor criteria
Hypomelanotic macules	Confetti skin lesion
Angiofibroma	Dental enamel pits
Ungual fibroma	Intraoral fibromas
Shagreen patch	Retinal achromic patch
Multiple retinal hamartomas	Multiple renal cysts
Multiple cortical tubers and/ or radial migration lines	Nonrenal hamartomas
Subependymal nodules	Sclerotic bone lesion
Subependymal giant cell astrocytoma	
Cardiac rhabdomyoma	
Lymphangiomyomatosis	
Angiomyolipomas	

Hypomelanotic macule occurs in approximately 90% of TS cases, angiofibroma in 75%, shagreen patch in approximately 50% of cases, ungual fibroma in 20%, and confetti skin lesion in 3%–58% of cases. Forehead plaque is seen in 25% of TSC patients and is paired with angiofibroma in the diagnostic criteria.^2^This patient had both multiple facial angiofibromas and multiple forehead plaques.

Approximately half of the patients with TS have normal intelligence, seizures account in about 63%–78% of infants with TSC, and facial angiofibromas occur in about 75% of patients, with onset typically occurring between ages 2 and 5 years.^1^, ^2^, ^3^Authors discovered epilepsy in 71.2% of adult TSC patients in a study.^6^ The classic triad of mental retardation, epilepsy, and facial angiofibroma, also known as the Vogt triad, occurs in only 29% of the cases, with 6% lacking all three of them.^1^, ^5^ Hence, it can be related to our patient, who has no history of epilepsy and has normal intelligence.

Brain findings in TSC comprise of multiple cortical tubers observed in 90%, which are commonly seen together with radial migration lines, subependymal nodules (SEN), and subependymal giant cell astrocytomas (SEGA), which are observed in 80% and 5%–15%, respectively. SEN is often detected prenatally or at birth, and SEGA is likely to arise during childhood or adolescence but unlikely after 20 years of age.^2^ Calcified multiple subcortical tubers with radial migration lines and subependymal nodules were seen in our patient as well.

In regards to renal findings, three major renal manifestations of TSC are as follows: angiomyolipoma, cysts, and renal cell carcinoma.^2^, ^7^ Out of these three, only angiomyolipoma has been considered a part of major diagnostic criteria. Angiomyolipomas are benign tumors composed of varying proportions of vascular, smooth muscle, and adipose tissue. It is to be noted that a patient who has lymphangiomatosis (LAM) and renal angiomyolipoma but no other features of TSC does not meet criteria for a definite diagnosis.^2^, ^3^

Angiomyolipomas are relatively specific for TSC with an incidence of 50%–75%; however, they do occur in the general population as well with an incidence of 1%–2% and a female predominance of 6:1. Classic angiomyolipoma occurs mostly in females in the fifth decade, sporadically, while angiomyolipoma in TSC presents earlier in the third decade with no sex predominance. Angiomyolipoma in TSC is more likely to be multiple (97% vs. 13%), bilateral (80% vs. 12%), grow with time (67% vs. 21%), and bleeding tendency (44% vs. 14%).^8^, ^9^ The number and size of angiomyolipomas are known to increase with age, as evidenced by the second‐hit hypothesis and are more common in adolescence, with a higher frequency in girls. Since the patient in this report had her first presentation at the age of 27 years, the increase in size and number could not be estimated. However, she had multiple such lesions of variable size, which could have been increasing for a long time.^10^, ^11^

Symptoms of angiomyolipoma are absent or minimal, with most complaints being painless hematuria, as in our patient, flank pain, or a gross retroperitoneal bleed. There are some other complications that have been reported, which include Wunderlich syndrome and end‐stage renal disease (ESRD). Wunderlich syndrome is spontaneous bleeding that is confined to the subcapsular and perirenal spaces and can arise when an enlarging vessel becomes aneurysmal and ruptures within an angiomyolipoma. Our patient did not have the classical Lenk's triad of the syndrome, which consists of acute flank pain, palpable flank mass, and hypovolemic shock. However, the imaging findings do demonstrate an aneurysmal vessel and hematoma.^12^, ^13^ The incidence of ESRD in TSC is low and that can be ascertained in our patient, who shows no signs of renal failure despite the presence of multiple angiolipoma in each kidney.^12^, ^14^

In relation to the imaging finding for diagnosis, the presence of focal or diffuse fat containing masses is the characteristic feature of an angiomyolipoma, which can be visualized with all forms of ultrasonography, CT, and MRI with the latter being the best alternative in case of limitations or equivocal findings with other modalities.^15^ It is needless to say the biopsy should not be the mode of diagnosis due to the vascularity of the tumor and increased morbidity.^8^ In our case, CT was done, and it was able to delineate the lesion accurately for us to be able to diagnose the condition.

Regarding treatment of renal angiomyolipoma, recommendations are to use both tumor size and symptomatology to assess the course of tumor progression and the type of intervention to be used. According to one study, lesions smaller than 4 cm were less likely to be symptomatic than those larger than 4 cm (24% vs. 52%), and they were also less likely to grow (27% vs. 46%).^16^ Surgical interventions in angiomyolipoma are reserved for patients with symptomatic lesions or lesions with rapid growth. Our patient had symptoms of hematuria, for which she underwent cystoscopy with the evacuation of hematoma from the bladder; however, surgical management of the condition was declined.

Renal manifestations of TSC were the second leading cause of premature death after severe intellectual disability, so it is recommended that patients are to be closely monitored and treated as soon as possible.^2^, ^3^

Angiomyolipoma has been found not only in the kidney but also in other organs, including the liver as seen in this patient.^17^ The incidence of hepatic angiolipoma is increasing in part due to routine monitoring of patients with TS and renal angiomyolipoma.^2^ Hepatic angiomyolipomas are usually asymptomatic, though one study mentioned spontaneous hemorrhage from a hepatic angiomyolipoma in a patient with TS.^18^, ^19^The clinical significance of recognition of hepatic angiomyolipoma lies in the fact that misdiagnosis of these usually benign lesions may result in unnecessary and invasive diagnostic procedures.^20^

Other findings associated with TS, such as LAM, cardiac rhabdomyoma, dental and ophthalmologic findings, and other skin findings along with associated clinical manifestations, were not evident in our patient. However, counseling regarding the conditions, possible manifestations in the future and treatment was given to the patient.

## 3 CONCLUSIONS

Tuberous sclerosis is a rare neurocutaneous disorder. Sometimes a patient may appear with life‐threatening symptoms or may not have any symptoms at all for an extended period of time. This case of TS with hematuria as the only symptom that manifested was exceptional and can be regarded as a unique addition to the literature.

## AUTHOR CONTRIBUTIONS

**Gauri Adhikari:** Conceptualization; writing – original draft; writing – review and editing. **Prabin Pandey:** Data curation; investigation; visualization. **Kishor Bhattarai:** Data curation; methodology; resources; writing – original draft. **Chhabi Khadka:** Data curation; investigation; software; supervision; writing – review and editing. **Gopal Adhikari:** Methodology; writing – original draft; writing – review and editing.

## FUNDING INFORMATION

No source of funding was available for the report.

## CONFLICT OF INTEREST

The authors declares no conflict of interests.

## ETHICAL APPROVAL

The study was conducted in accordance with the Declaration of Helsinki. The paper exempts from ethics committee approval as only one case was reported with the consent from the patient.

## CONSENT

Written informed consent was obtained from the patient to publish this report in accordance with the journal's patient consent policy.

## Data Availability

Data sharing is not applicable to the article as no new data were created or analyzed in this study.
